# Amelioration of Acute Kidney Injury in Lipopolysaccharide-Induced Systemic Inflammatory Response Syndrome by an Aldose Reductase Inhibitor, Fidarestat

**DOI:** 10.1371/journal.pone.0030134

**Published:** 2012-01-12

**Authors:** Kazunori Takahashi, Hiroki Mizukami, Kosuke Kamata, Wataru Inaba, Noriaki Kato, Chihiro Hibi, Soroku Yagihashi

**Affiliations:** 1 Department of Pathology and Molecular Medicine, Hirosaki University Graduate School of Medicine, Hirosaki, Japan; 2 Sanwa Kagaku Kenkyusho, Nagoya, Japan; French National Centre for Scientific Research, France

## Abstract

**Background:**

Systemic inflammatory response syndrome is a fatal disease because of multiple organ failure. Acute kidney injury is a serious complication of systemic inflammatory response syndrome and its genesis is still unclear posing a difficulty for an effective treatment. Aldose reductase (AR) inhibitor is recently found to suppress lipopolysaccharide (LPS)-induced cardiac failure and its lethality. We studied the effects of AR inhibitor on LPS-induced acute kidney injury and its mechanism.

**Methods:**

Mice were injected with LPS and the effects of AR inhibitor (Fidarestat 32 mg/kg) before or after LPS injection were examined for the mortality, severity of renal failure and kidney pathology. Serum concentrations of cytokines (interleukin-1β, interleukin-6, monocyte chemotactic protein-1 and tumor necrosis factor-α) and their mRNA expressions in the lung, liver, spleen and kidney were measured. We also evaluated polyol metabolites in the kidney.

**Results:**

Mortality rate within 72 hours was significantly less in LPS-injected mice treated with AR inhibitor both before (29%) and after LPS injection (40%) than untreated mice (90%). LPS-injected mice showed marked increases in blood urea nitrogen, creatinine and cytokines, and AR inhibitor treatment suppressed the changes. LPS-induced acute kidney injury was associated with vacuolar degeneration and apoptosis of renal tubular cells as well as infiltration of neutrophils and macrophages. With improvement of such pathological findings, AR inhibitor treatment suppressed the elevation of cytokine mRNA levels in multiple organs and renal sorbitol accumulation.

**Conclusion:**

AR inhibitor treatment ameliorated LPS-induced acute kidney injury, resulting in the lowered mortality.

## Introduction

Systemic inflammatory response syndrome (SIRS) caused by sepsis is a life-threatening disease accompanied by multiple organ failure. The combination of acute renal failure and sepsis is associated with a 70% mortality while mortality rate of acute renal failure alone is 45% [**1]**, [2****]. Although early commencement of broad antibiotic treatment may provide some possibilities of recovery from this condition, it often results in irreversible organ damage and fatal condition [**3]**, [4****]. Renal failure caused by acute kidney injury is one of the major reasons for the mortality. Currently, there is no effective treatment for this serious condition in part due to complex mechanisms of how SIRS leads to acute kidney injury. It is therefore essential to elucidate the precise processes or explore the measures involved in development of acute kidney injury for the effective treatment.

Lipopolysaccharide (LPS) is known to induce SIRS condition in animal models and widely exploited for the search for mechanisms of SIRS-related conditions and exploration of drug development [**5]**, [6****]. In previous studies, LPS was shown to cause experimentally SIRS with acute renal failure. In this setting, production of cytokines and nitric oxide, sympathetic dysregulation, alterations of immunity as well as proinflammatory conditions were proposed to contribute to the induction of acute renal failure [**7]**, [8****]. It remains unknown, however, whether the acute kidney injury is caused by excessive cytokines or other products secreted from damaged cells or inflammatory cells.

Polyol pathway has long been studied for its role in the pathogenesis of diabetic complications [**9]**–[11****] and recently on the implication of ischemia/reperfusion injury [**12]**–[15****]. The major regulating enzyme, termed aldose reductase (AR), converts glucose to sorbitol, which in turn changes into fructose by the enzyme of sorbitol dehydrogenase. When this pathway is activated, coenzyme nicotinamide adenine dinucleotide phosphate (NADPH) is so consumed as to decrease nitric oxide and glutathione reductase, resulting in enhancement of oxidative stress. The process is now considered to attribute to various organ damages encountered in ischemic injury, transplantation or trauma [**16]**, [17****]. In our previous studies, acute renal failure induced by hindlimb ischemia was successfully rescued by early intervention with AR inhibitor which prevented the pathological lesions of acute kidney injury [**17]**, [18****]. In this study, we extended our search to examine whether the acute kidney injury in LPS-induced SIRS can be influenced by AR inhibitor and explored its mechanism.

## Materials and Methods

### Animals

Male C57Bl/6J mice (Japan Clea Inc., Tokyo, Japan), 8–12 weeks of age, were used in this study. They were reared in air-conditioned with 6 am to 18 pm light cycle and fed standard rodent chow ad libitum. LPS (*Escherichia coli* 0111:B4)(Sigma-Aldrich, Milwaukee, WI, USA) was freshly dissolved in sterile pyogen-free water each time when applied. First, mice were injected intraperitoneally with LPS (16 mg/kg) and followed for 72 hours to see the survival rate. The dose of LPS was determined by preliminary experiments that demonstrated longer survival than 24 hours in a half of the animals injected. To examine the inhibitory effects of AR inhibitor, groups of animals were orally (by gavages) given AR inhibitor (Fidarestat)(32 mg/kg)(Sanwa Kagaku Kenkyusho, Nagoya, Japan) dissolved in N-methyl-D-glucamine buffer (Sigma, St.Louis, MO, USA) just 1 hour before or 15 minutes after LPS injection. The dose of AR inhibitor was determined by the previous data that showed an effective survival in mice with hindlimb-ischemia-induced renal failure [**18**]. Control mice were given buffer alone. For examination of the effects of AR inhibitor on the renal damage, mice were injected with lower dose of LPS (8 mg/kg) and killed at the point of 5 hours and 20 hours under deep anesthesia with pentobarbital. Minimal amount of blood was withdrawn from the heart and kidneys were collected for the biochemical and structural investigations. Lung, liver and spleen were also harvested for the comparison of mRNA expressions of cytokines with those in the kidney. Since animals injected with LPS underwent oligo- or anuria, urine analysis was not performed.

All the experiments were carried out in strict accordance with the recommendations in the Guide for the Care and Use of Laboratory Animals of the National Institutes of Health. The protocol was approved by the Committee on the Ethics of Animal Experimentation of Hirosaki University (Approval Number #80-2000). All efforts were made to minimize suffering of animals.

### Laboratory data and enzyme-linked immunosorbent assay (ELISA) of cytokines

Blood urea nitrogen and creatinine were examined by an autoanalyzer (SpotChem EZ, SP4430, Arkray, Edina, MN, USA). Blood concentrations of cytokines were examined by commercially available Endogen® Mouse ELISA kit (Pierce Biotechnology, Inc. Rockford, IL, USA) for interleukin (IL)-1β, IL-6, monocyte chemotactic protein (MCP-1), and tumor necrosis factor (TNF)-α. The procedures for the measurement were followed the methods described in the manufacturer's protocol. The plates were analyzed at 450 nm and 540 nm using EL340 microplate bio kinetics reader (Bio-Tek Instruments, Winooski, VT, USA). Samples whose concentration fell outside the standard curve were further diluted and the test repeated.

### Cytokine mRNA transcripts

After extirpation of kidney, renal tissues were homogenized for the analysis of mRNA transcript expression of cytokines. Tissues of lung, liver and spleen were also homogenized in a similar manner. In brief, dissected tissues were homogenized and dissolved in Isogen (Wako Pure Chemical, Osaka, Japan) and 0.2 ml of chloroform was added. Samples were mixed thoroughly and centrifuged at 15,000 rpm for 15 min. The upper aqueous phase was transferred and the RNA was precipitated by the addition of 0.5 ml of 2-propanol followed by 10 min incubation at room temperature and centrifugation at 15,000 rpm for 10 min. The precipitate was washed with 75% ethanol, and the final pellet was resuspended in RNase free water. The RNA concentration was assessed using DU530 Life Science UV/Vis Spectrophotometer (Beckman Coulter Inc., Brea, CA, USA) prior to processing for real-time PCR (RT-PCR) analysis. To remove any traces of DNA, samples were treated with DNase I (Invitrogen, Carlsbad, CA, USA) for 15 minutes. RNA (2 µg) was reverse transcribed into cDNA using Superscript VILO™ cDNA synthesis kit (Invitrogen) following the manufacturer's protocol. Then RNase H (Invitrogen) was added to remove RNA from cDNA. Commercially available primer and probe sets (Gene expressions assays, Applied Biosystems, Carlsbad, CA, USA) for target genes of IL-1β, IL-6, inducible nitric oxide synthase (iNOS), MCP-1, toll-like receptor 4 (TLR4), TNF-α and internal standard of glyceraldehyde-3-phosphate dehydrogenase (GAPDH) were mixed with cDNA and Thunderbird Probe qPCR Mix (Toyobo, Osaka, Japan). Then the mixture (25 µl) was loaded onto an optical reaction plate in duplicate. An established RT-PCR assay using the relative quantification method (ddCT) was conducted in ABI PRISM 7000 Sequence Detection System (Applied Biosystems, Foster City, CA, USA).

### AR expressions and polyol metabolites in the kidney

For the detection of AR protein expression, renal cortex and medulla were homogenized in Tris-saline-acid (TSA)- phenylmethylsulfonyl fluoride (PMSF) buffer (pH 8.0) and centrifuged (15,000 rpm). SDS-PAGE was performed using the Xcell SureLock system (Invitrogen) in the reducing condition. Aliquots of 100 µg samples of protein were dissolved in the same sample buffer [2.5% 2-mercaptoethanol, 62.5 mmol Tris–HCl, 10% glycerol, 2% SDS, 0.0025% bromophenol blue, and 50 mmol reducing agent (dithiothreitol; DTT), pH 6.8] and loaded onto the NuPAGE®4-12% Bis-Tris Gel (Invitrogen). After completion of the migration, the proteins were transferred to a polyvinylidene fluoride membrane (Immobilon-P; Millipore, Bellerica, MA, USA) in a transfer buffer (25 mmol Tris, 0.2 mol glycine, and 20% methanol) using a wet transfer unit of the iBlot™ Dry Blotting system (Invitrogen). The membrane was blocked with 5% milk in TBS buffer with 0.1% Tween 20 for 1 hour, and then reacted with antibody to mouse AR and β-actin specific antibody (Santa Cruz Biotechnology Inc., Santa Cruz, CA, USA). A final incubation was carried out with peroxidase-conjugated anti-goat IgG (Santa Cruz) for 60 min at room temperature. Immunodetection was performed by enhanced chemiluminescence (Amersham-Pharmacia, Buckinghamshire, UK) and exposed to imaging films.

Sorbitol and fructose contents in whole renal homogenates were measured by liquid chromatography with tandem mass spectrometry (LC/MS/MS) methods described previously [**18**]. The concentrations were expressed as nanomoles per milligrams per protein.

### Pathological investigations

Renal cortical tissues were fixed in 10% buffered formalin and embedded in paraffin. 4 µm-thick sections were applied to hematoxylin-eosin staining, histochemical staining with naphthol-AS-D chloroacetate esterase for detection of neutrophils, immunohistochemical staining for detection of macrophages, and ApopTag® staining for detection of apoptotic cells.

For immunohistochemical staining, sections of formalin-fixed tissues were deparaffinized and pretreated with methanol containing 0.3% H_2_O_2_ to eliminate endogenous peroxidase activity. For the detection of macrophages, immunostains using monoclonal antibodies to Iba-1 (Wako Pure Chemical, Osaka, Japan) were used. Following the application of first antibodies, the sections were incubated with secondary and tertiary agents using a streptavidin-biotin-peroxidase detection kit (Histofine SAB-PO Kit, Nichirei, Tokyo). N, N'-diaminobenzidine was used to visualize peroxidase deposition at the antigenic sites, and these sections were lightly counterstained with hematoxylin. The specificity was confirmed by the replacement of the primary antibodies with non-immune sera or by the omission of the primary antibodies. To detect apoptosis, we adopted staining with ApopTag® kit (Millipore, Bedford, MA, USA) following the manufacturer's protocol.

To express the changes in an objective manner, cortical areas were subjected to analysis of evaluation of vacuolar degeneration of tubular cells. When more than 50% of tubular cells were vacuolar, the change was scored as 3+, 20%≤∼<50% as moderate, 2+, 5%≤∼<20% as mild, 1+, and nil, <5%. Neutrophil counts were determined as number per unit area in cortex on sections stained with naphthol-AS-D-chloroacetate esterase. The number of cells stained positive with ApopTag® were also counted on a single cross section at a magnification of x200 in each animal and expressed as a percentage of positive cells to total nucleated tubular cells counted per unit area. In contrast, macrophages stained positive with Iba-1 were counted on the 3-5 frames of cortical area and expressed as a number of cells per unit area. For the objective comparison of the staining results among all the groups, renal sections from each group were randomly mounted on a single slide (i.e., 4 renal tissues on one slide) and stained under the same condition.

### Statistical analysis

All the quantitative values are expressed as mean±SE as a representative of a group. Statistical differences among groups were examined by analysis of variance with post-hoc Bonferroni corrections. p-Values less than 0.05 were considered to be significant. Survival time was estimated using the Kaplan–Meier method. Log-rank test was used to compare survival times between groups. p-Values less than 0.05 were considered to be significant.

## Results

### Survival analysis

**Figure 1:**
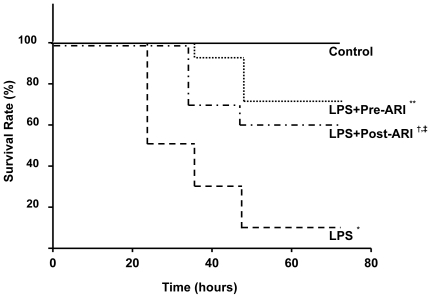
Temporal changes of mortality rate of experimental mice. Most of lipopolysaccharide (LPS)-treated animals (9/10; 90%) died during 72 hours after LPS injection (n = 10). When the animals were treated with aldose reductase inhibitor (ARI) before (n = 14) or after LPS injection (n = 10), death rate was significantly suppressed to 29% and 40%, respectively. Although there was a trend toward a better survival in pretreated group compared to posttreated group, the difference was not significant at the endpoint. Each group consists of 8∼14 animals. *p<0.01 vs Control, **p<0.01 vs LPS, †p<0.05 vs LPS, ‡p<0.05 vs Control.

The mortality rate of the mice injected with LPS was 90% (9/10 animals) in untreated animals at the point of 72 hours after injection of LPS **(** **Figure 1** **)**. When the LPS-injected animals were given orally AR inhibitor 32 mg/kg before LPS injection, the mortality rate was significantly reduced to 29% (4/14) (p<0.001 vs untreated LPS-treated animals). The reduction of mortality rate was also gained in mice given AR inhibitor after the injection of LPS (40%, 4/10). The difference in the mortality rate between untreated and AR inhibitor-treated groups of either pretreatment or posttreatment was significant (p<0.01 for both). However, there was no significant difference between pretreatment and posttreatment, although there was a trend for better survival in the former.

### Laboratory data and cytokine levels

**Figure 2:**
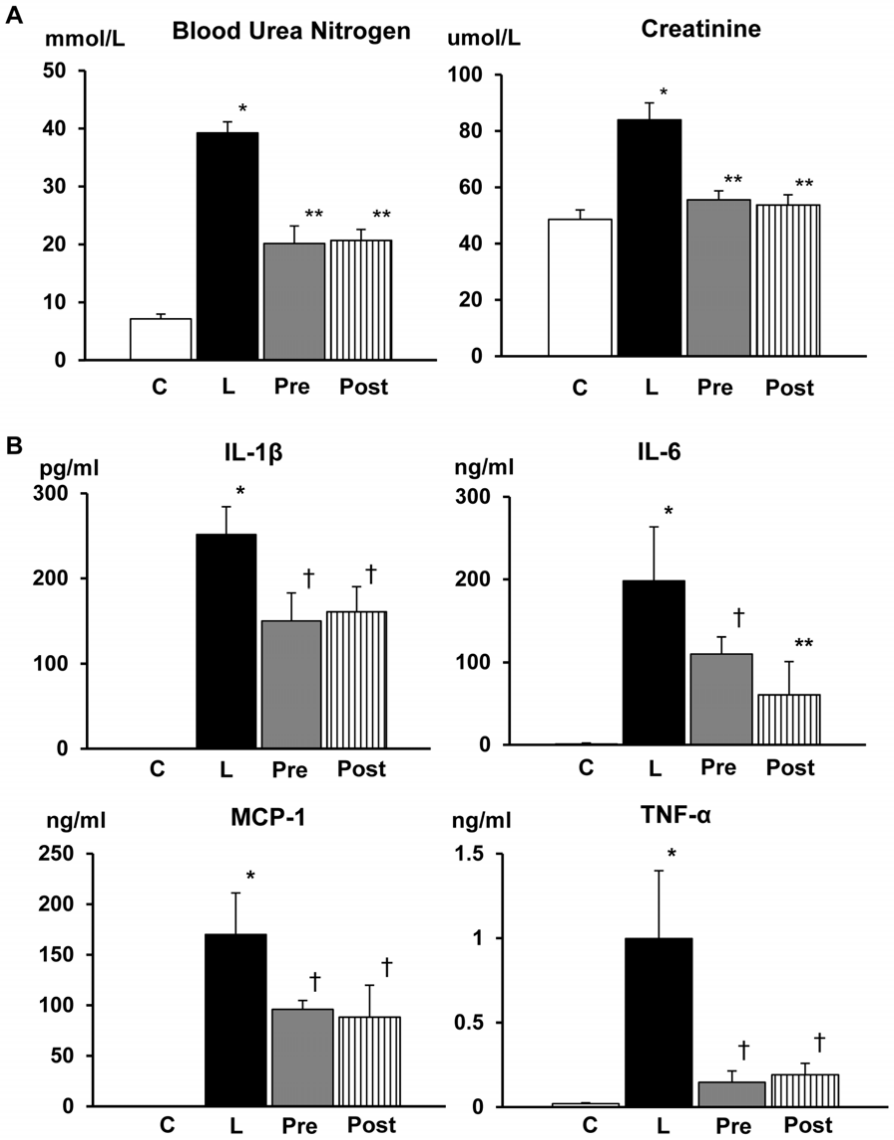
Laboratory data and cytokine levels in the blood. A: Marked elevation of blood urea nitrogen (BUN) and creatinine in the blood was detected in lipopolysacchride (LPS)-injected mice, and treatment with aldose reductase inhibitor (ARI) either before or after LPS injection significantly suppressed to 50% for BUN and to nearly normal for creatinine. There was no difference in the values between ARI pretreatment and posttreatment. B: Serum concentrations of interleukin (IL)-1β, IL-6, monocyte chemotactic protein (MCP)-1 and tumor necrosis factor (TNF)-α were all markedly increased in LPS-injected mice, while they were undetectable in LPS-free mice. Treatment with ARI either before or after the LPS injection significantly suppressed the elevations of IL-1β, IL-6, and MCP-1 to about 50% whereas TNF-α elevation was repressed to almost to normal. There were no significant differences in the values of any cytokines between pretreatment and posttreatment. Each group consists of 6∼8 animals. Bar stands for mean ± SE. C: Control, L: LPS-injected group, Pre: LPS+ARI-pretreatment group, Post: LPS+ARI-posttreatment group. *p<0.01 vs Control, **p<0.01 vs LPS, †p<0.05 vs LPS.

Blood urea nitrogen values of LPS-injected mice were significantly elevated more than 7 times of untreated mice **(** **Figure 2A** **)**. In mice treated with AR inhibitor before and after the injection of LPS, the rise of blood urea nitrogen was suppressed to about 50% of untreated animals. Similarly, creatinine values were significantly (1.8 fold) elevated in LPS-treated animals and either pre- or posttreatment with AR inhibitor improved the values to nearly normal levels.

Serum concentrations of IL-1β, IL-6, MCP-1 and TNF-α were all markedly increased in LPS-injected mice, while they were undetectable in LPS-free mice **(** **Figure 2B** **)**. Treatment with AR inhibitor either before or after LPS-injection significantly suppressed these elevations to about 50% for IL-1β, IL-6 and MCP-1, whereas those of TNF-α and reverted almost normal. There were no significant differences in the values of any cytokines between pretreatment and posttreatment.

### Cytokine mRNA expressions

**Figure 3:**
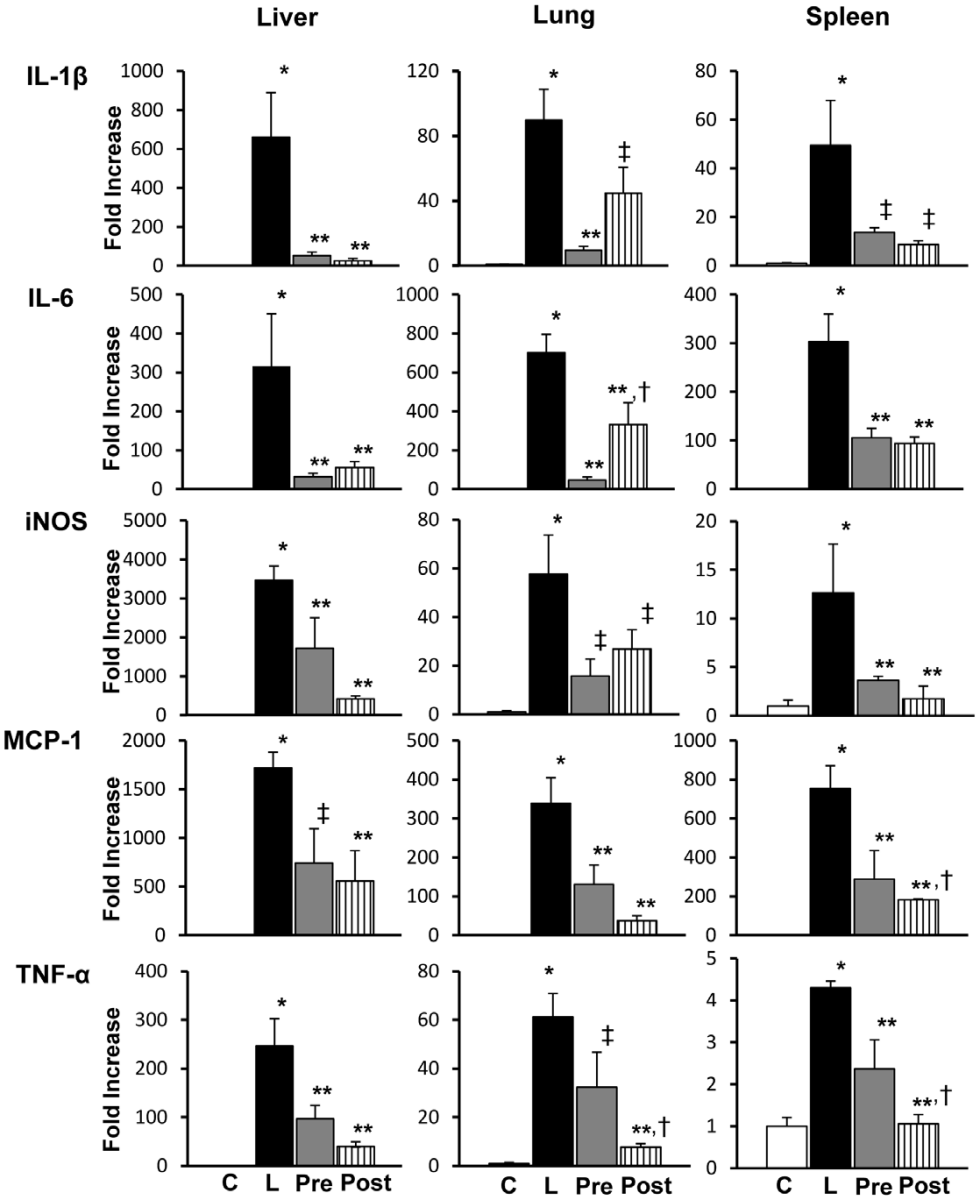
Cytokine mRNA expressions in liver, lung and spleen. Tissues from the liver, lung and spleen in experimental mice at 5 hours after lipopolysaccharide (LPS) injection showed increased mRNA expressions of interleukin (IL)-1β, IL-6, inducible nitric oxide synthase (iNOS), monocyte chemotactic protein (MCP)-1 and tumor necrosis factor (TNF)-α in LPS-injected mice. Treatment with aldose reductase inhibitor (ARI) either before or after the injection of LPS significantly suppressed the elevations of all the cytokines. The alterations were all similar in the liver, lung and spleen. Overall, there was no significant difference in mRNA expressions between pretreated and posttreated groups except for IL-6, TNF-α in the lung and MCP-1 and TNF-α in the spleen (see text). Each group consists of 6∼8 animals. Bar stands for mean ± SE. C: Control, L: LPS-injected group, Pre: LPS+ARI-pretreatment group, Post: LPS+ARI-posttreatment group. *p<0.01 vs Control, **p<0.01 vs LPS, †p<0.05 vs LPS+ARI-pretreatment group, ‡p<0.05 vs LPS.

Cytokine mRNA expressions were examined by RT-PCR on the lung, liver spleen, and kidney tissues obtained from mice with or without LPS-injection **(** **Figure 3** **)**. At 5 hours after the LPS-injection, mRNA expressions of the cytokines such as IL-1β, IL-6, iNOS, MCP-1 as well as TNF-α were all elevated in the liver, lung and spleen. AR inhibitor treatment either before or after LPS-injection caused significant suppression of mRNA expressions of these cytokines. While the extent of suppression by AR inhibitor treatment was comparable in most of the cytokines in a given organ between pretreatment and posttreatment, mRNA expression of IL-6 in the lung was more potently suppressed in pretreatment group compared to posttreatment group. In contrast, MCP-1 in the spleen and TNF-α in the lung and spleen were more potently suppressed in posttreatment group compared to pretreatment group. The significant elevation of cytokine mRNA expressions in the liver, lung and spleen disappeared in the samples obtained from the mice at 20 hours after LPS-injection (data not shown).

**Figure 4:**
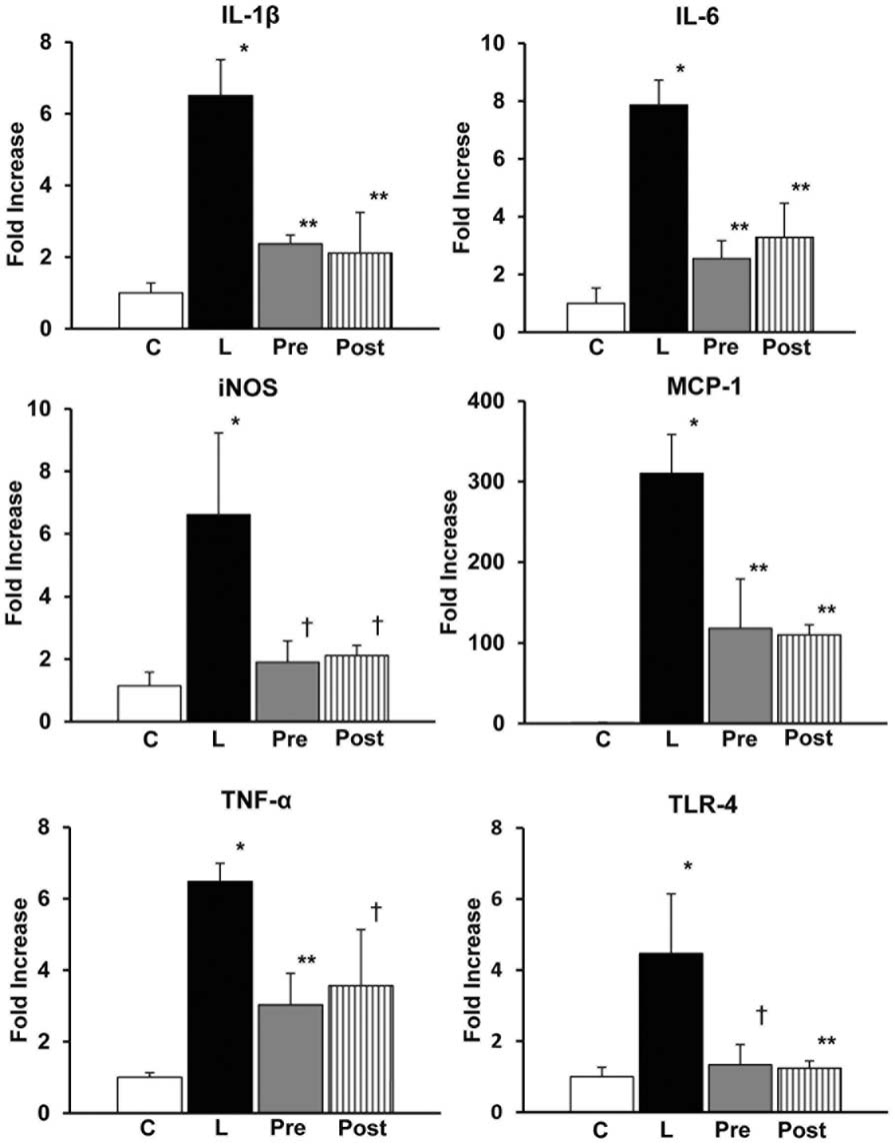
Cytokine mRNA expressions in kidney. There were marked elevations of mRNA expressions of interleukin (IL)-1β, IL-6, inducible nitric oxide synthase (iNOS), tumor necrosis factor (TNF)-α and toll like receptor (TLR)4 to about 4∼8 fold, and of monocyte chemotactic protein (MCP)-1 to about 300 fold in the renal cortex in the lipopolysaccharide (LPS)-injected mice taken at 20 hours after LPS-injection. Treatment with aldose reductase inhibitor (ARI) before or after the injection of LPS significantly all suppressed the elevations. There was no significant difference between pretreated and posttreated groups. Each group consists of 5∼8 animals. Bar stands for mean ± SE. C: Control, L: LPS-injected group, Pre: LPS+ARI-pretreatment group, Post: LPS+ARI-posttreatment group. *p<0.01 vs Control, **p<0.01 vs LPS, †p<0.05 vs LPS.

Cytokine mRNA expressions in the kidney at 5 hours after the LPS-injection were not elevated (data not shown). In contrast, mRNA expressions of IL-1β, IL-6, iNOS, TNF-α and TLR4 in the kidney were elevated all about 4∼8 fold, whereas those of MCP-1 were elevated about 300 fold at 20 hours after the LPS injection. Treatment with AR inhibitor either before or after LPS-injection significantly suppressed the elevation **(** **Figure 4** **)**. There was no significant difference in the levels of mRNA expressions of these cytokines between pretreatment and posttreatment.

### AR expression and polyol metabolites in the kidney

**Figure 5:**
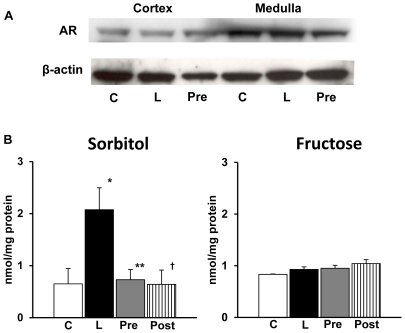
Western blot analysis of aldose reductase (AR) and polyol concentrations in kidney. A; Western blot analysis showed strong expression of murine AR in both renal cortex and medulla. There was no alteration of AR expression in the kidney of mice injected with lipopolysaccharide (LPS) and treatment with AR inhibitor (ARI) did not influence on the expressions. The expression of AR was stronger in medulla compared to cortex. B: There was a marked elevation of sorbitol concentration in LPS-injected mice. ARI treatment both before and after LPS injection significantly suppressed this elevation. There was no difference between pretreated and posttreated groups. Each group consists of 6 animals. Bar stands for mean ± SE. *p<0.01 vs Control, **p<0.01 vs LPS, †p<0.05 vs LPS C: Control, L: LPS-injected group, Pre: LPS+ARI-pretreatment group, Post: LPS+ARI-posttreatment group.

Western blot analysis showed strong expression of murine AR in both renal cortex and medulla **(** **Figure 5A** **)**. The latter contained more but there was no alteration in the AR expressions in the kidney of mice injected with LPS **(** **Figure 5A** **)**. AR inhibitor treatment did not influence on the expressions.

Sorbitol concentrations were markedly increased in the renal homogenates in LPS-injected mice **(** **Figure 5B** **)**. The increase was normalized in AR inhibitor-treated groups of both pretreatment and posttreatment. In contrast, there was no significant alteration in fructose concentrations in LPS-injected mice and AR inhibitor treatment did not influence the values.

### Renal pathology

**Figure 6:**
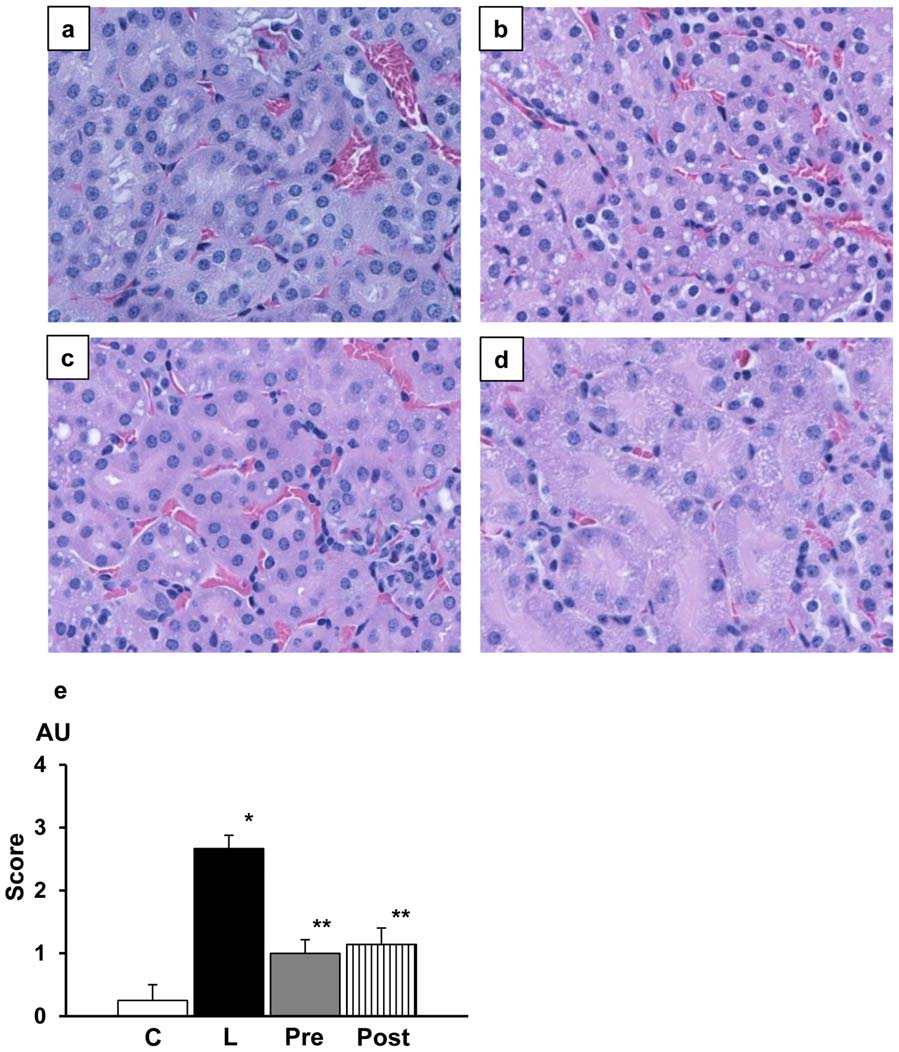
Renal pathology in experimental mice stained with hematoxylin and eosin. Compared to lipopolysacchrade (LPS)-free mice (a), marked vacuolar degeneration of tubular cells were noted in LPS-injected mice (b). Such vacuolar changes were markedly suppressed in either pretreated group (c) or posttreated group (d). Quantitative estimation of the degenerated cells confirmed the effects of aldose reductase inhibitor (e). Magnification of the pictures was all x320. Each group consists of 6 animals. Bar stands for mean ± SE. AU; arbitrary unit C: Control, L: LPS-injected group, Pre: LPS+ARI-pretreatment group, Post: LPS+ARI-posttreatment group. *p<0.01 vs Control, **p<0.01 vs LPS.

**Figure 7:**
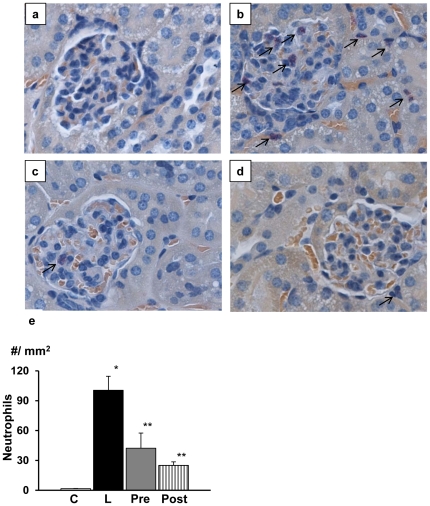
Neutrophilic infiltration in the renal cortex. On naphthol AS-D chloroacetate esterase stained sections, there was no apparent infiltration of neutrophils in untreated mice (a). Lipopolysaccharide (LPS)-injection caused severe infiltration of neutrophils in the glomeruli and interstitium (b) and this change was suppressed by treatment with aldose reductase inhibitor (ARI) (c and d). Quantitation of neutrophil infiltration revealed significant increase in the number of neutrophils in LPS-injected mice and ARI treatment suppressed the infiltration of neutrophils (e). There was no significant difference between pretreated and posttreated groups. Magnification of the pictures was all x400. Each group consists of 6 animals. Bar stands for mean ± SE. C: Control, L: LPS-injected group, Pre: LPS+ARI-pretreatment group, Post: LPS+ARI-posttreatment group. *p<0.01 vs Control, **p<0.05 vs LPS.

**Figure 8:**
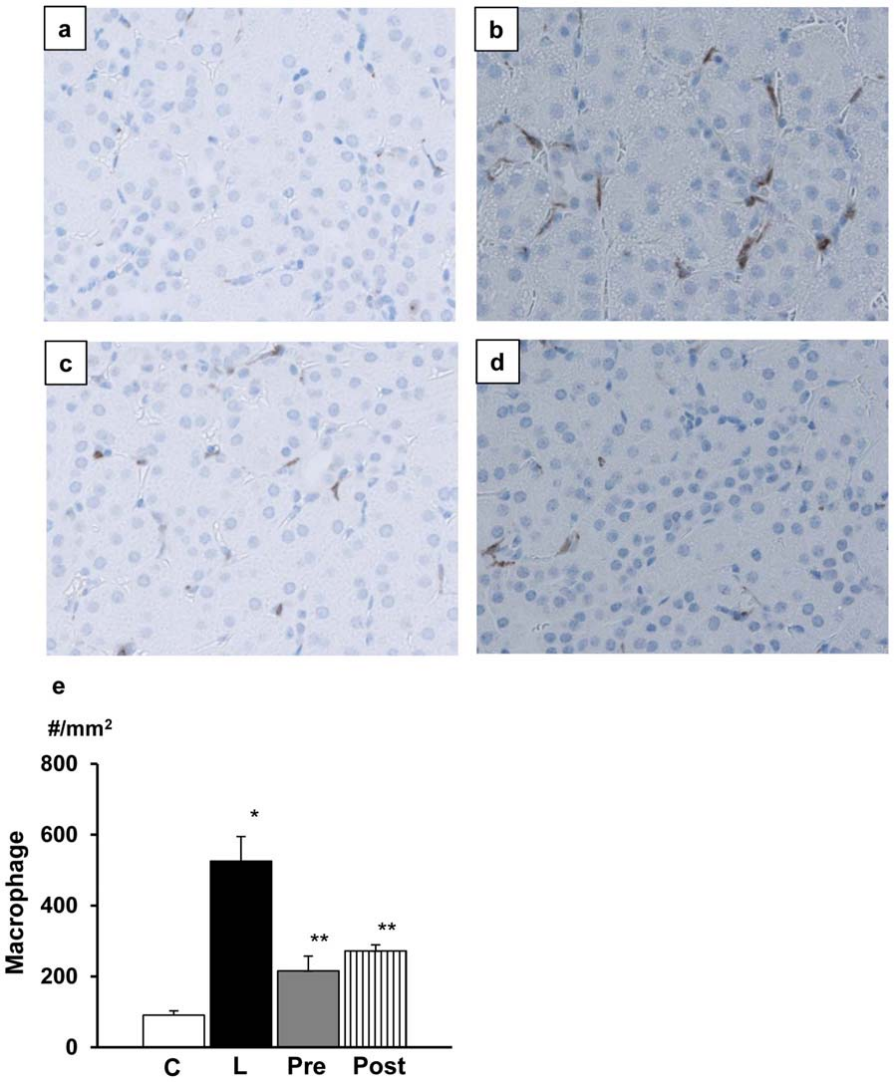
Macrophage migration in renal cortex. In lipopolysassharide (LPS)-free mice, macrophages were hardly found (a). In contrast, there was marked infiltration of macrophages immunostained with Iba-1 in the interstitium (b). The infiltration was inhibited by treatment with aldose reductase inhibitor (ARI) either before or after LPS injection (c and d). Quantitative evaluation confirmed the findings. There was no significant difference between pretreated and posttreated groups. Magnification of the pictures was all x320. Each group consists of 6 animals. Bar stands for mean ± SE. C: Control, L: LPS-injected group, Pre: LPS+ARI-pretreatment group, Post: LPS+ARI-posttreatment group. *p<0.01 vs Control, **p<0.05 vs LPS.

**Figure 9:**
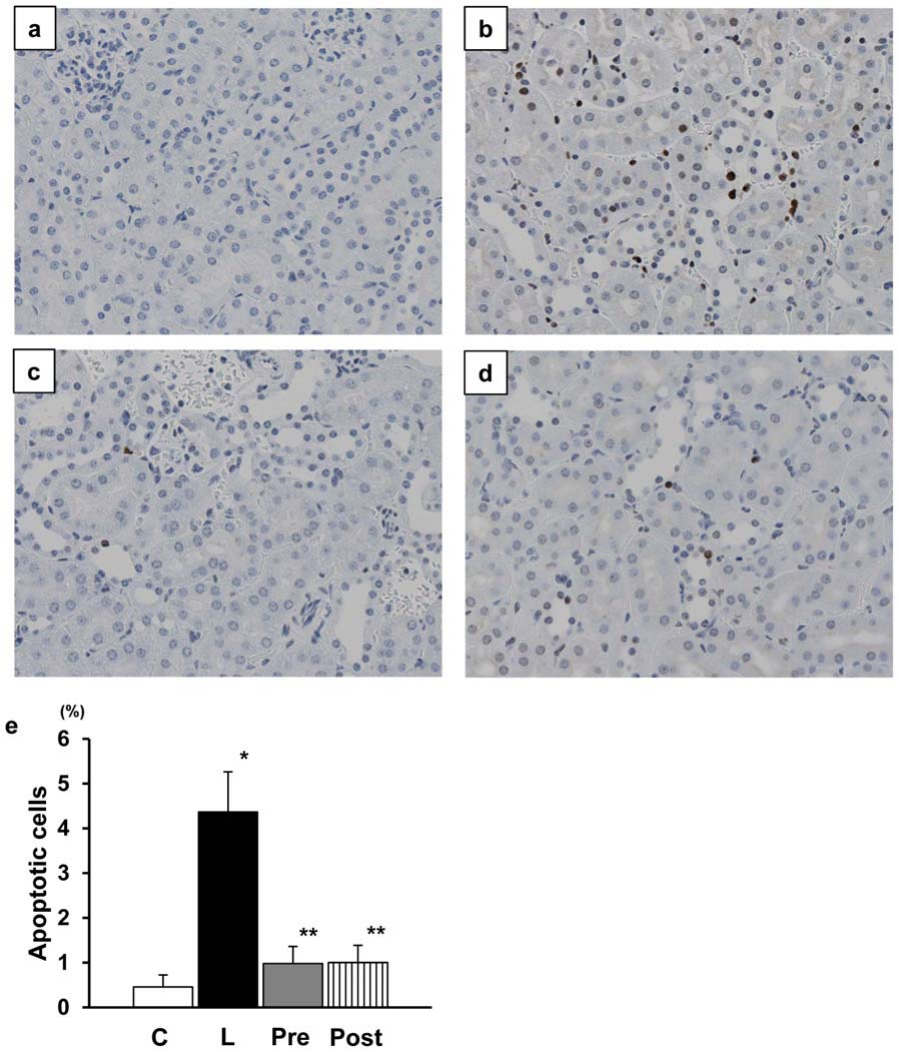
Apoptotic renal tubular cells stained with ApopTag®. While there was no apparent positive reaction in lipopolysaccharide (LPS)-free mice (a), the LPS injected mice showed a marked increase in apoptotic cells (b) and treatment with aldose reductase inhibitor (ARI) significantly suppressed the appearance of positive cells (c and d). Quantitation confirmed the above findings (e). There was no significant difference between pretreated and posttreated groups (x240). Each group consists of 6 animals. Bar stands for mean ± SE. C: Control, L: LPS-injected group, Pre: LPS+ARI-pretreatment group, Post: LPS+ARI-posttreatment group. *p<0.01 vs Control, **p<0.05 vs LPS.

On hematoxylin eosin-stained sections, renal tubular cells underwent significant vacuolar degenerative changes **(** **Figure 6** **)**. AR inhibitor treatment either before or after LPS injection improved the degenerative changes. There was no significant difference in the effects between pretreatment and posttreatment. Concurrently, there appeared neutrophilic infiltration in the glomeruli and interstitium of the kidney in LPS-injected mice **(** **Figure 7** **)**. AR inhibitor treatment again ameliorated the neutrophil infiltration and the effects were comparable between pretreated and posttreated group. Similarly, there was strong infiltration of macrophages in the interstitium in LPS-injected mice, which was suppressed by AR inhibitor treatment **(** **Figure 8** **)**. Finally, consistent with the degenerative changes of tubular cells, there was a marked increase in apoptotic cells of tubular cells in LPS-injected mice **(** **Figure 9** **)**. ARI treatment suppressed the appearance of apoptotic cells and the difference in the effects of AR inhibitor between pretreatment and posttreatment was negligible.

## Discussion

In this study, acute kidney injury induced by LPS-injection was prevented by treatment with AR inhibitor. Either prior to or after the administration of LPS, a single dose of AR inhibitor suppressed the mortality rate of the animals. The blood concentrations of blood urea nitrogen and creatinine and the enhanced mRNA expressions of cytokines in the kidney of LPS-injected animals were all inhibited by treatment with AR inhibitor. Although pretreatment effects on the mortality appeared to be slightly more potent compared to posttreatment, there was no significant statistical difference. The vacuolar degeneration and apoptosis of renal tubular cells were inhibited by AR inhibitor which also suppressed infiltration of neutrophils and macrophages. In one previous study that demonstrated the suppressive effects of AR inhibitor on LPS-induced mortality, the mortality was proposed to be ascribed to the effects on LPS-induced cardiac failure and information of the kidney was not provided [**19**]. Acute renal failure is another serious sequel of LPS-induced SIRS condition [**1]**, [2****] and its patho-physiological background is recently called as acute kidney injury [**20]**, [21****]. This is the first to demonstrate that acute kidney injury elicited by LPS-induced endotoxemia associated with cytokinemia was significantly ameliorated by AR inhibitor.

We searched for possible mechanisms that may underlie the mechanism of how AR inhibitor suppressed the process of acute kidney injury and the mortality in LPS-injected animals. As previously demonstrated in mice with LPS-induced cardiac failure, inflammatory signaling mediated by transcription factors (NF-κB and AP-1) and stress-activated mitogen activated protein (MAP) kinases were augmented to enhance cytokine production in endotoxemia [**22**] and AR inhibitor effectively reduced such signaling and thereby suppressed cytokine production. Our study also demonstrated the increased production of renal cytokines and blood concentrations of blood urea nitrogen and creatinine in LPS-injected mice. We confirmed that treatment with AR inhibitor significantly suppressed the damage to the renal tissues compared to those in untreated group. In the setting of LPS-induced exdotoxemia, macrophages were shown to be activated to enhance AR expression and its activity with production of cytokines [**7]**, [8], [23****]. In the ischemic kidney, macrophages in response to tissue damage migrate in the injured sites shortly after neutrophilic infiltration mediated by chemokine receptor signals [**24]**, [25****]. These cells in turn produce cytokines of IL-1β, IL-6, IL-12 and TNF-α. Our findings that the lung, liver and spleen tissues rich in macrophages were all exerted to produce cytokines in LPS-injected mice are in accord with the data in the uvea or lung in other studies [**26]**, [27****]. In contrast, the involvement of AR in the kidney related to macrophage migration was not addressed in previous studies. Induction of iNOS by LPS may also be responsible for systemic vasodilation via release of nitric oxide and glomerular microthrombi due to endothelial damage [**2]**, [7], [8****], resulting in acute renal failure, to which AR inhibitor counteracted.

It should be of note that cytokine mRNA expressions were expressed differentially at a different time among the organs involved. In the kidney, cytokine induction started later compared with the lung, liver and spleen rich in macrophages, indicating late effects on the kidney. The magnitude of increases in cytokine mRNA was much smaller in the kidney than that in other organs in LPS-injected animals. Concentrations of blood urea nitrogen and creatinine were already increased 5 hours after LPS-injection before the rise of tissue cytokine mRNA expressions. From these findings, it is likely that mechanisms other than the cytokine production may also be involved in the genesis of LPS-induced acute kidney injury. Indeed, the cytokines are not always correlated with the severity of acute kidney injury in myonephropathic-metabolic syndrome [**28**].

Acute renal vasoconstriction is considered to be an initial trigger for sepsis-induced acute renal failure [**29]**, [30****]. Ischemia-reperfusion is well known to cause acute kidney injury. In fact, LPS-induced endotoxemia rapidly causes renal vasoconstriction and ischemic tissue damage [**31**], while evidence supporting the involvement of both innate and adaptive immunity in ischemia-reperfusion-induced acute kidney injury has accumulated in recent years [**21]**, [32****]. In ischemia- reperfusion-induced acute kidney injury, renal tubular cells become scaffold for complement binding and upregulate toll-like receptors (TLRs), which lead to cytokine/chemokine production [**33]**, [34****]. Molecules such as high-mobility group B1 (HMGB1), heat shock protein, hyaluronan, biglycan released from damaged tissues also activate TLRs and lead to downstream activation of transcription factors that regulate the expression of survival genes or proinflammatory cytokines and chemokines [**35]**, [36****]. Neutrophilic accumulates are the hallmark of renal ischemia- reperfusion injury in mouse models [**21**]. In this setting, leukocytes, renal endothelial cells promote inflammation after ischemia-reperfusion by increasing adhesion molecule expression and vascular permeability. As such, there is a possibility that AR inhibitor may have ameliorated LPS-induced acute kidney injury in a manner similar to the protection from the ischemia-reperfusion-induced renal damage.

Beneficial effects of AR inhibitor on ischemia-reperfusion injury have repeatedly been demonstrated in the heart [**13]**, [37****], brain [**38**], and retina [**14**] by using AR overexpressing mice as well as AR deficient mice. In these studies, ischemic tissue injury augmented polyol flux resulting in redox changes that directly altered JAK-STAT pathways and protein kinase C activation with excessive oxygen radical damage [**12]**, [13], [16****]. AR inhibitor treatment effectively reduced the infarction size in the heart and brain as well as retinal tissue damage in those studies. In agreement with their findings we also found increased apoptotic cells in the kidney and AR inhibitor effectively suppressed the changes. Involvement of AR was also pointed out in hindlimb ischemia-induced renal failure [**18**]. In this study, acute kidney injury caused by hindlimb ischemia was associated with excessive cytokine production which was prevented by AR inhibitor treatment. Renal tubular cells regulates cell osmolarity through induction of osmotic response element (ORE) gene linked with AR gene which is a promoter to control cell integrity to accommodate to osmotic stress [**15]**, [39****]. ORE is also responsive to be activated by TNF-α or nitric oxide [**40]**, [41****]. When cells are activated by osmotic stress, they develop vacuolation with swollen mitochondria, resulting in apoptotic cell death [**9]**, [39****]. In keeping with this story, in our study, marked accumulation of sorbitol was detected in the kidney of LPS-injected mice, indicating the presence of osmotic stress. AR inhibitor significantly inhibited the sorbitol accumulation and tubular cell vacuolation together with the inhibition of apoptosis. From these findings, the treatment with AR inhibitor may be protective for preservation of renal tubular cells at least in part directly by inhibiting polyol pathway activation. It should be of note, however, that there was no significant increase in fructose concentrations in renal tissues in LPS-injected mice. Since the kidney samples were taken shortly after LPS injection (20 hours), it might have been too early to see significant increase in the fructose concentrations. Alternatively, high permeability of fructose in contrast to the stable intracellular sorbitol may account for this unexpected finding. More detailed analysis on the time course changes of polyol flux as well as the expression of sorbitol dehydrogenase should be required to solve this question in future studies.

In summary, our current study demonstrated the beneficial effects of AR inhibitor for the protection of acute kidney injury induced by LPS. The results indicated that AR plays a crucial role in the acute kidney injury and early treatment with AR inhibitor should seriously be considered for future clinical application for this serious disorder.
